# Anaesthesia in Veterinary Oncology: The Effects of Surgery, Volatile and Intravenous Anaesthetics on the Immune System and Tumour Spread

**DOI:** 10.3390/ani13213392

**Published:** 2023-11-01

**Authors:** Ana Vidal Pinheiro, Gonçalo N. Petrucci, Amândio Dourado, Isabel Pires

**Affiliations:** 1 Department of Veterinary Sciences, School of Agricultural and Veterinary Sciences (ECAV), University of Trás-os-Montes e Alto Douro (UTAD), 5000-801 Vila Real, Portugal; amandio.dourado@onevetgroup.pt (A.D.); ipires@utad.pt (I.P.); 2 Onevetgroup Hospital Veterinário do Porto (HVP), 4250-475 Porto, Portugal; goncalopetrucci@gmail.com; 3 Center for Investigation Vasco da Gama (CIVG), Department of Veterinary Sciences, Vasco da Gama University School (EUVG), 3020-210 Coimbra, Portugal; 4 CECAV—Veterinary and Animal Research Center, University of Trás-os-Montes and Alto Douro, 5001-801 Vila Real, Portugal

**Keywords:** perioperative period, surgery, immune system, tumours, intravenous anaesthetics, volatile anaesthetics, veterinary oncology

## Abstract

**Simple Summary:**

Anaesthesia plays an important role in diagnosis procedures and the treatment and pain management of oncological patients. However, studies suggest that anaesthetic drugs may increase the risk of tumour dissemination in the perioperative period by directly and indirectly suppressing the immune system, which is primarily responsible for controlling tumour growth. Science faces the challenge of understanding their influence on the immune system to develop anaesthetic strategies that assure greater immune stability in patients with cancer or other immune-compromising diseases.

**Abstract:**

Throughout the course of oncological disease, the majority of patients require surgical, anaesthetic and analgesic intervention. However, during the perioperative period, anaesthetic agents and techniques, surgical tissue trauma, adjuvant drugs for local pain and inflammation and other non-pharmacological factors, such as blood transfusions, hydration, temperature and nutrition, may influence the prognosis of the disease. These factors significantly impact the oncologic patient’s immune response, which is the primary barrier to tumour progress, promoting a window of vulnerability for its dissemination and recurrence. More research is required to ascertain which anaesthetics and techniques have immunoprotective and anti-tumour effects, which will contribute to developing novel anaesthetic strategies in veterinary medicine.

## 1. Introduction

The growing attention paid to animal health [1] and the development of novel diagnostic and therapeutic procedures have significantly impacted the lifespan of companion animals [2]. Malignant tumours tend to be more prevalent in older animals [3,4], which can be linked to various age-related factors such as alterations in cell-mediated immunity and the phenomenon of immunosenescence [5]. The diagnosis of cancer in dogs and cats has increased significantly in recent years, making it one of the most prevalent causes of death. [6,7]. This fact has led to novel therapeutic oncology approaches, which are considered based on the tumour line and the patient’s health [8]. Surgical excision is still considered the gold standard for treating solid tumours [9]; however, even in tumour resections with histologically clean margins, an undetected residual risk of the intra-surgical dissemination of tumour cells still exists [10]. For this reason, it is often required to combine surgical excision with other treatment alternatives, such as chemotherapy, radiation or biological therapies [11,12,13,14].

Considering human and animal studies, the main risk of tumour dissemination occurs in the perioperative period [15,16], during which cancer cells and the immune system are influenced by stress, pain [17,18], anaesthesia [19] and surgical procedures [20]. These factors induce a stress response that starts a cascade of physiological mechanisms beneficial to the restoration of homeostasis but also favourable to tumour growth [20], which include immunomodulatory and pro-tumour effects such as immune suppression and tumour angiogenesis [17,18]. Therefore, the perioperative period can be seen as a window of opportunity to minimise the pro-tumour and anti-immune mechanisms triggered by surgical stress and anaesthetic drugs, suggesting new clinical approaches with neurophysiological and immune preservation. Alternative surgical and anaesthetic procedures, such as minimally invasive surgery [21], regional anaesthesia [22], opioid-free anaesthesia [23], total intravenous anaesthesia [24] and multimodal analgesia [25,26], are gaining significance in clinical practice, with particular emphasis on oncological patients due to their immunoprotective effects.

According to this knowledge, this review aims to debate the influence of anaesthetic drugs on the immune system and tumour cells based on retrospective studies in humans and animals.

## 2. Search Methodology

This review has been conducted to describe the outcomes of relevant experimental studies. A search strategy of the PubMed database was conducted to find the qualifying articles. We applied the following keywords: “anaesthetic strategies in oncological surgery”; “anaesthesia and cancer”; “anaesthesia and cancer recurrence and metastasis”; “veterinary anaesthesia oncology”; “cancer in anaesthesia dogs”; “anaesthesia and cancer recurrence and metastasis in dogs”; “regional anaesthesia in animals”; “total intravenous anaesthesia in small animals”; “intravenous anaesthetics and tumour spread”; “volatile anaesthetics and tumour spread”; “surgery and immune system”; “surgery and tumour dissemination”; “perioperative period and tumour spread”; “cancer and immune system”. The search terms determined were full text, ten years, randomised controlled trial, clinical trial, meta-analysis, review and veterinary anaesthesia.

## 3. The Immune System as the Main Player in the Tumour’s Defence

The tumour growth and spread process is influenced by the tumour’s characteristics but mainly by the host’s immune system [8]. The scientific community recognises that a high number and high activity of natural killer cells and cytotoxic T lymphocytes correlate with enhanced anti-tumour immune responses and the elimination of neoplastic cells from blood circulation [27,28]. Likewise, dendritic cells (DCs) are an essential subpopulation of leukocytes for inducing and regulating an adaptive anti-tumour immune response [29]. Cancer immunoediting is part of a dynamic concept that explains the role of the immune system and immune cells in malignant transformation, emphasising cancer immunotherapy [30].

In veterinary and human searches, a high count and ratio of circulating leukocytes (CD4+ and CD8+ T cells, dendritic cells, macrophages and regulatory T cells) [31,32], as well as their presence in the tumour microenvironment [33,34], may be associated with the prognosis of certain solid tumours. In felines with mammary carcinoma, the neutrophil-to-lymphocyte ratio has been suggested as a prognostic factor. In contrast, high numbers of total leukocyte counts, neutrophils, and the neutrophil/lymphocyte ratio were associated with an increased risk of tumour-related death [31]. Moreover, the lymphocyte/monocyte ratio can predict the prognosis of newly diagnosed canines with diffuse large B-cell lymphoma in terms of time progression and lymphoma-specific survival [32] and the histopathological grade of canine mast cell tumours [35]. In canine osteosarcoma, monocyte and lymphocyte counts were used as prognostic indicators [36]. Other studies observed that in dogs with oncological disease, the percentage of Th1 was considerably lower and Th2 was significantly higher compared to healthy dogs [37]. In addition, they determined that dogs with metastases had even lower Th1 values than ill dogs without metastases [37]. Researchers observed a correlation between tumour-infiltrating lymphocytes and histopathological characteristics in canines with mammary carcinoma, suggesting that a significant quantity of lymphocytes infiltrating mammary carcinoma is associated with lymphatic invasion and a high histopathological grade [38]. Furthermore, the tumour-infiltrating lymphocytes proved indispensable for tumour growth and their spread potential [38]. In the review “A role for T-lymphocytes in human breast cancer and in canine mammary tumours”, the authors compare tumour T-lymphocyte infiltration and the CD4+/CD8+ T-cell ratio with low survival rates, the action of Th2 cells in the acceleration of tumour progression and a poor prognosis with the presence of large numbers of Treg cells [39]. In human breast cancer, the role of macrophages in tumour progression was observed as a facilitator of the invasion of tumour cells [40] due to their plasticity and ability to adapt to the different physiological conditions that the body presents, which promote the production of cytokines type II, anti-inflammatory responses, and pro-tumour functions [41]. In pancreatic and colorectal human cancers, elevated Th2 levels and an imbalance of the Th1/Th2 ratio lead to an increase in pro-inflammatory interleukins that promote the progression of the disease [42,43].

Considering the immune system as a main player in the body’s defence against tumour growth, some human and animal studies suggest a new approach to minimising the impact on the immune cells of several suppressive factors such as stress, pain, anaesthesia and surgery [19,44,45]. According to studies in dogs, the administration of recombinant canine interferon (rCaIFN-γ) before, during and after anaesthesia may reduce the suppression of natural killer (NK) activity, inhibit intraoperative cancer cell dissemination and prevent postoperative cancer recurrence and metastasis [46,47]. In humans, it has been demonstrated that the administration of nonsteroidal anti-inflammatory agents to oncological patients has anti-tumour properties by inhibiting the pro-tumour activity of the enzyme COX-2 of macrophages and increasing the activity of T regulatory cells [48,49], both of which have been shown to promote tumour progression in humans. Dendritic cells also play an important role in immunotherapies, eliciting Th1 and Th17 cell differentiation [50] and infiltrating solid malignancies [51].

## 4. Tumour Surgery as a “Starting Point” for Tumour Progression

Excisional tumour surgery is the main approach for treating primary solid tumours [9] and the major facilitator of metastatic cell dissemination [52]. Since the eighteenth century, this condition has been researched as a “starting point” for accelerating tumour progression [53]. Since then, several studies have been published on the relationship between surgery and cancer progression to understand this biological behaviour, including 1. the dissemination of tumour cells via the manipulation of the neoplastic tissue [54]; 2. the effect of surgery as a window of opportunity for the development and spread of tumour cells [55]; 3. the change in the state of tumour dormancy via surgical tumour excision [56] and decreased antiangiogenic factor secretion [57].

First, the surgical manipulation of neoplastic tissue promotes the release of neoplastic cells into the blood and lymphatic system [54], where they seek pre-metastatic niches to initiate a new cell growth cycle [58]. Besides that, before surgery, most oncology patients already have cancer cells in their circulatory system, which does not necessarily indicate the presence of metastases or tumour recurrence [59,60]. In human research, less than 0.01% of circulating tumour cells are successful in tumour metastasis, depending on the immune response and the tumour cells’ ability to avoid the defence cells [61]. Nonetheless, human investigations have demonstrated a correlation between tumour cells in peripheral blood after surgery and poor prognosis prediction [62].

**Figure 1:**
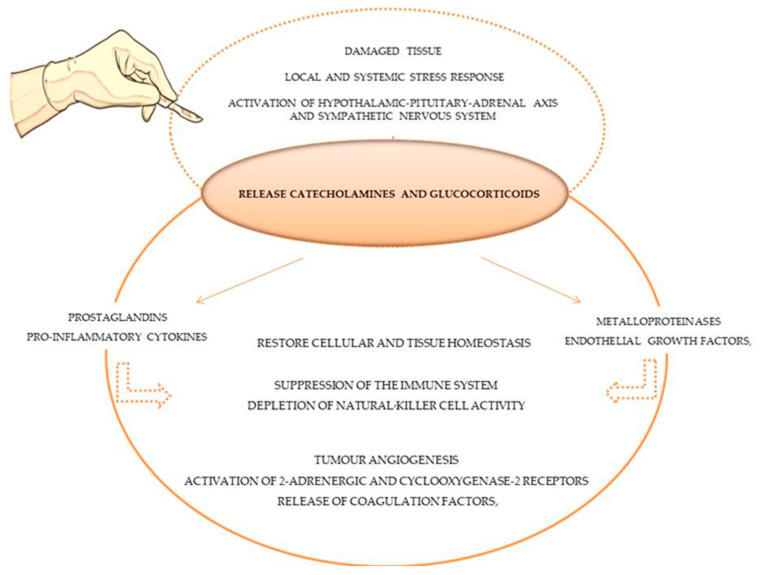
Stress and inflammatory response of tissue surgery damage in immune system and tumour cells.

Secondly, the microenvironment of the damaged tissue in the area of the surgical procedure changes, and minutes after the incision, a proportional local and systemic stress response is generated [20]. Physiological mechanisms such as local inflammation, sympathetic nervous system activation, and the hypothalamic–pituitary–adrenal axis (HPA) are activated to restore cellular and tissue homeostasis following tissue injury [20], as demonstrated in Figure 1. In response, immunomodulatory chemicals are released, specifically catecholamines and glucocorticoids, which induce prostaglandins (PGE2), pro-inflammatory cytokines (e.g., IL-10), metalloproteinases and endothelial growth factor secretion [17]. These immunomodulatory effects can result in a suppression of the immune system, producing direct and indirect pro-tumour effects such as tumour angiogenesis, the activation of 2-adrenergic and cyclooxygenase-2 receptors, the release of coagulation factors, and the depletion of natural killer cell (lymphocytes CD3- and CD56+) activity [18], which plays a significant role in anti-tumour immunity by controlling the dissemination of cancer cells [27,28]. Platelets and coagulation factors are released to reestablish haemostasis, forming protective aggregates that shield tumour cells from natural killer cells [63]. In actuality, this biological process, which simultaneously attempts to restore homeostasis, modifies the immunological, metabolic, endocrinological, neuronal, inflammatory and immune microenvironments, thereby fostering tumour growth and dissemination [18].

Considering the inflammatory, immunosuppressive and tumour-spreading effects of surgery trauma, the potential advantages of minimally invasive surgery (MIS) over conventional open surgery have been discussed in human and animal studies, but with inconsistent results [21,64]. MIS seems to offer certain benefits, including reduced tissue inflammation and immune suppression, as well as less blood loss, decreased postoperative pain and reduced hospitalisation [21,64,65]. However, longer surgical and anaesthesia times and an increased risk of technical complications, such as wound dehiscence and local inflammation, could potentially be disadvantages when compared to open surgery [56,66,67].

Lastly, tumour dormancy is a condition characterised by the presence of tumour cells in the absence of evidence of oncological disease [68]. This phenomenon may explain both cancer recurrences after long periods of remission and the maintenance of reduced neoplastic cells after adjuvant oncological therapies [69], as a result of the absence of angiogenic competence, a homeostatic equilibrium between tumour cells and the immune response or the presence of an environment that does not promote tumour growth [68]. Surgical tumour excision helps cancer survive by altering the biological characteristics of neoplastic cells, such as proliferation, apoptosis, metastasis, and dormancy condition [70], and blood levels of tumour growth promoters (e.g., IL-6, TNF-α, VEGF) are higher after surgery, whereas antiangiogenic mediators such as endostatin and angiostatin are undetectable [71]. This finding supports the hypothesis that surgery and neuroendocrine mediators that control angiogenesis and inflammation can “awaken” a dormant tumour condition and dormant micrometastases [72]. As well, tumour angiogenic activity, which is the physiological process of generating new blood vessels from existing ones, is essential for the survival and development of tumour cells, including their growth, invasion and spread [73].

Even though some biological factors may paradoxically enhance the proliferation and dissemination of metastatic cells, surgical tumour removal provides an evident benefit in preventing the growth and spread of cancer [9]. Furthermore, both animal and human studies have demonstrated that the use of a less traumatic surgical technique and pharmaceutical substances, such as COX-2 selective nonsteroidal anti-inflammatory drugs [74,75], antithrombotics [63] and antiangiogenics [76,77], can make these biological effects simpler to control.

## 5. The Impact of the General Anaesthetics on the Immune System and Their “Anti-” and “Pro-Tumoral” Effects

**Figure 2:**
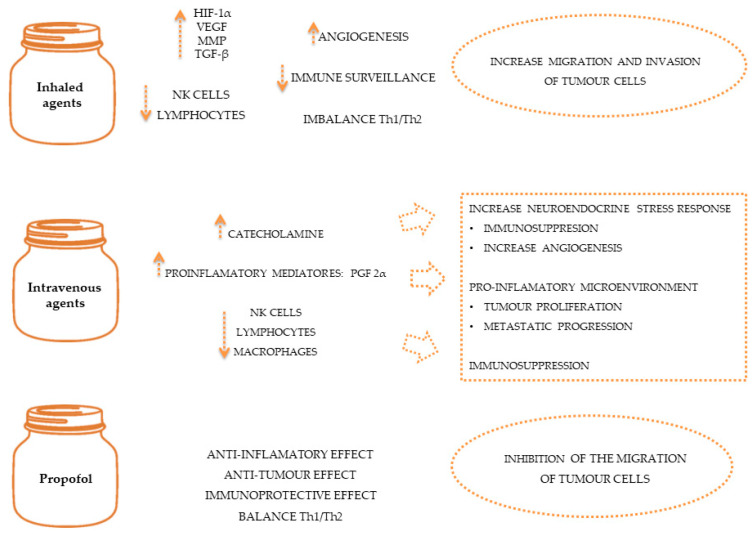
The effect of anaesthetics on immune system and on tumour spread.

Oncological diseases often require the use of anaesthetic and analgesic agents during diagnostic [78,79], therapeutic or palliative procedures to control pain [80,81] and minimise the effects of the stress response [76]. General anaesthetics include volatile agents like isoflurane and intravenous agents like propofol, which have been linked to immunomodulatory properties such as the inhibition of NK cell activity [82] and pro-tumour effects such as the inhibition of prostaglandin synthesis by tumour cells [47]. Due to their immunosuppressive effects, volatile anaesthesia may be associated with poorer outcomes than intravenous anaesthesia [83,84], as demonstrated in Figure 2. However, human studies suggest that general anaesthetics in combination with regional anaesthesia can reduce the inflammatory response and endothelial permeability, preventing the spread of cancer cells [65]. Also, studies in dogs and cats suggest that loco-regional therapy serves to reduce the quantity of intra and post-operative opioids, allowing their adverse effects to be reduced [85,86,87].

### 5.1. Volatile Anaesthetic Agents

Volatile anaesthetics, such as isuflurane and sevoflurane, are halogenated agents used to induce and maintain general anaesthesia through the respiratory system [66] as well as regulate anaesthetic depth in real time, being useful in both short and long anaesthesia [88]. Depending on whether they are used solo or in combination, they can exhibit more or fewer effects on tumour cells and on the immune system [82]. In human research, volatile anaesthetic agents promote dose- and time-dependent immune suppression [89], inhibit lymphocyte proliferation [90] and reduce the function of natural killer (NK) cells, which play a crucial role in protecting against the spread of cancer cells [91]. Moreover, they affect the biological functions of macrophages, neutrophils and natural killer (NK) cells by enhancing anti-apoptosis pathway signalling [92] and modulating intracellular signals of cancer cell activity, such as proliferation, migration, invasion and chemoresistance [93,94]. Animal studies suggest that volatile anaesthetics suppress the immune system more than propofol [24], inhibit the cytotoxic activity of natural killer cells [82], reduce the secretion of TNF and IL-1 [95] and, when combined with propofol, maintain similar ratios of CD3+/anti-B cells and CD4+/CD8+ cells before and after anaesthesia [24].

Isuflurane is an inhaled anaesthetic agent commonly used in veterinary medicine that produces a state of unconsciousness due to its action on the central nervous system and without analgesic properties [96]. Like other inhalation anaesthetics of this type, it suppresses the respiratory and cardiovascular systems, is absorbed by inhalation and is rapidly distributed by tissues, including the brain [96]. Its absorption, distribution and elimination by the lungs are quick, with the clinical consequences of rapid induction and recovery and easy and rapid control of the depth of anaesthesia [96]. Several human studies have investigated the immunomodulatory effects of isuflurane, with results that may be less beneficial for cancer patients due to the fact that it suppresses the immune system significantly. Therefore, they suggest that isuflurane may lower the number of circulating NK cells [97], inhibit their activity [98] and reduce their ability to respond to INF-g stimulation [99]. They can also diminish the number of B lymphocytes, IFN-y, IFN-α, TNF-α and IL-2 circulated [97] as well as induce the apoptosis of B and T lymphocytes [98]. When compared to intravenous anaesthesia, isuflurane can more significantly diminish the ratio of Th1/Th2 lymphocytes [98], with no significant changes in CD4+ cells (helper T cells) [100]. General anaesthesia only with isuflurane can increase the levels of HIF-1α. However, this does not happen when it is associated with propofol, which is correlated with a reduction in cancer cells’ malignant activity [92]. Their anti-inflammatory properties are induced by suppressing the NF-kB pathway [101], which regulates essential cellular processes including inflammatory responses, cellular growth and apoptosis, which are associated with certain conditions such as cancer [102]. Studies in vitro demonstrate that isuflurane can promote chemotherapeutic resistance in colon cancer cells [103] and enhance renal cancer growth and malignant potential [104]. Isuflurane also promotes the growth and migration of glioblastoma cells [105], increases the malignant potential of ovarian cancer cells through the up-regulation of markers associated with the cell cycle, proliferation, and angiogenesis [106] and enhances the proliferation of cervical cancer cells through the upregulation of histone [94]. Immune suppression and its effects on oncological patients were also observed in studies on animals anaesthetised with isuflurane, suggesting that this inhalant agent suppress the immune system more than propofol [24], inhibits the cytotoxic activity of natural killer cells [82] and decreases the secretion of TNF-α and IL-1 [95]. This effect may be associated with tumour metastasis or recurrence in the post-operative period [82]. In horses, isuflurane reduces the activity of the neutrophil myeloperoxidase desgranulation system [107], which contributes to the elimination of injured host tissues and invading microorganisms [108]. In dogs, it was shown that the combination of isuflurane with propofol can affect lymphocyte counts, which decreased considerably 24 h after anaesthesia, and the ratios of CD3+/anti-B cells and CD4+/CD8+ cells did not significantly differ before and after anaesthesia [24]. In mice, a significant increase in melanoma growth was observed when they were exposed to isuflurane [109].

Sevoflurane is an inhaled anaesthetic agent used to induce and maintain anaesthesia [96]. It produces a relatively quick and smooth start to anaesthesia in dogs, followed by an effective recovery [96]. Vomiting, decreased blood pressure, increased breathing rate, muscle tension, excitement, small muscle contractions and a temporary inability to breathe are the most common adverse effects of sevoflurane [96]. Human research has demonstrated that sevoflurane has the potential to inhibit NK cell activity, increase apoptosis in T and B lymphocyte cells [102] and suppress the release of IL-1β and TNF-α [90]. Both induce the dose-dependent inhibition of polymorphonuclear neutrophil apoptosis [110,111]. However, sevoflurane has no effect on the total lymphocyte count, decreasing the neutrophil and increasing the lymphocyte numbers [112]. Moreover, they promote the increased expression of HIF-1α [113] and inhibit the recruitment of macrophages [114]. Sevoflurane converts helper T cells into tumour-promoting T lymphocytes, decreasing the Th1/Th2 ratio [115]. Anaesthesia with sevoflurane may increase the levels of pro-tumour cytokines and metalloproteinases [116], have a systemic anti-inflammatory effect [117] and decrease hypoxia-induced growth and metastasis via suppressing HIF-1α [118]. They increase the survival of breast cancer cells via the modulation of intracellular Ca2+ homeostasis [93] and inhibit the viability, migration and invasion of osteosarcoma cells by inactivating the PI3K/ATK pathway [119], as well as the formation of lung cancer cells and metastasis by decreasing HIF-1α [95,120]. When combined with a spinal block, sevoflurane can limit the antitumor activity of liver mononuclear cells, probably by preserving the balance of Th1/Th2, preventing tumour spread [121]. In animal models, sevoflurane converts helper T cells into tumour-promoting T lymphocytes, decreasing the Th1/Th2 ratio which has been linked to the development and spread of canine cancer [37]. Moreover, sevoflurane plays an important role in regulating gene expression by inducing different mRNA expression [122], which is the primary molecular mechanism responsible for the pathological processes of human diseases, including cancer [123]. According to a rabbit study, the suppression of leukocyte subpopulations (CD45+, CD4+, CD8+, and CD21+ cells) increased by anaesthesia with sevoflurane alone or in combination with nitrous oxide [124]. On canine mammary tumour cells, sevoflurane at low concentrations demonstrated a significant increase in cell proliferation, whereas it was inhibited at high concentrations [125].

### 5.2. Intravenous Anaesthetic Agents

Propofol, alphaxalone and ketamine are all commonly used for the induction, co-induction and maintenance of anaesthesia [126,127,128]. They may be administered alone or in combination with other intravenous agents, volatile anaesthetics [92] or other techniques such as regional anaesthesia [129]. According to human and animal studies, certain intravenous agents induce protective immune effects, inhibiting tumour growth [24,130]. However, in vitro research with dog models did not reveal any effect of propofol, alphaxalone or ketamine on the cytotoxic activity of peripheral lymphocytes, albeit researchers acknowledge that these anaesthetics’ actions in a living organism could have different results [131]. Nonetheless, other studies have shown different findings, demonstrating that some of intravenous anaesthetic agents may promote the proliferation and migration of human breast cancer cell lines and oral squamous cell carcinoma [132,133].

Propofol (2,6-diisopropylphenol) is one of the intravenous anaesthetic agents routinely used in veterinary clinical practice for the induction and maintenance of general anaesthesia, with affinity for γ-aminobutyric acid (GABA) receptors and producing sedative and hypnotic effects [134]. They possess neuroprotective [135], anti-apoptotic [136] and anti-inflammatory actions in the central nervous system [137]. Metabolites of propofol inhibit the oxidant activities of neutrophils and myeloperoxidase and can have immunomodulatory effects during clinical use, particularly for the treatment of systemic diseases such as cancer [138]. Moreover, in human medicine, propofol is gaining emphasis in oncological surgery due to its “immunoprotective” [24] and “anti-tumoral” [130] properties, which are able to alter the biological characteristics of tumours by promoting the apoptosis of tumour cells, increasing tumour sensitivity to various chemotherapeutic agents [139] and decreasing the activity of tumour cells [92]. They also decrease the production of inflammatory mediators [140,141] and the immunity of the tumour microenvironment [142], modulating intrinsic processes such as neoangiogenesis [143]. In dogs anaesthetised with propofol, lower blood concentrations of catecholamines and cortisol have been demonstrated, suggesting less anaesthetic stress when compared to isuflurane [144]. Propofol inhibits the synthesis of prostaglandins by canine tumour cells and has no effect on the activity of lymphocytes and natural killer cells [47], as well as on the pro- and anti-inflammatory cytokines [24], reducing variations in the distribution of lymphocytes [145] and having no effect on the ratio of B/T cells and CD3+/CD8+ lymphocytes [82]. Moreover, they do not alter the balance of the Th1/Th2 lymphocyte ratio [100], which is associated with a slower progression and spread of tumour cells [37] and prevents the activation of HIF-1α by tissue hypoxia [92]. Dogs with diffuse large B-cell lymphoma anaesthetised with a volatile agent such as isuflurane or sevoflurane, opioids or propofol have a high risk of lymphoma relapse; however, the authors suggest that the results may have been caused by the opioids and inhalant agents [146]. In mice with breast cancer metastases, researchers compared the effects of propofol to those of ketamine, thiopental and halothane, suggesting that propofol has a small influence on NK cell function without promoting the growth of metastases [91]. In murine, a study suggests that propofol can directly suppress COX enzyme activity in peritoneal macrophages [147] and in human monocytes [148] which have been shown to promote tumour progression [49]. Studies in vitro with a rat model revealed that propofol inhibits tumour development by maintaining cytotoxic lymphocyte function [149]. A human study indicated that the risk of recurrence of oncological disease is substantially reduced when both propofol and epidural anaesthesia are used [150]. In addition, in humans, propofol-based anaesthesia during mastectomy is associated with increased survival compared to inhaled anaesthesia [150] and has an antitumor effect on rectal cancer by reducing the invasion capacity of this type of tumour cell [151]. In human lung cancer surgery, propofol maintains Th17/Treg cell balance through the GABA A receptor [152], which is important in regulating autoimmunity and cancer [153]. On the other hand, in vitro studies demonstrated that propofol promotes the migration and invasion of oral squamous cell carcinoma cells by upregulating the expression of the snail family transcriptional repressor 1 [132], which promotes tumour development in a variety of cancers and whose expression is related to the tumour grade and lymph node metastasis [154]. Using the Th1/Th2 ratio as an indicator, studies with patients undergoing craniotomy suggest that propofol can suppress the immune response induced by surgical stress [100].

Total intravenous anaesthesia (TIVA) is defined as the induction and maintenance of anaesthesia achieved exclusively by intravenous (IV) drugs [155]. This approach provides high hemodynamic stability, a smooth recovery and minimises exposure to inhalant anaesthetics [155]. Using propofol-based TIVA, a balanced anaesthesia approach is suggested in order to limit this agent’s dosage [156]. Propofol has advantageous pharmacokinetic properties for use in TIVA, including a quick onset, a brief duration of action and non-accumulative effects [157]. By combining different agents (anaesthetics, analgesics and/or sedatives) and anaesthetic techniques (loco-regional or neuraxial), it allows the reduction in each drug’s dose and its dose-dependent adverse effects while also delivering analgesia [129]. Immunoprotective effects, especially in T-lymphocytes and in pro- and anti-inflammatory cytokines, have been observed in dogs under general anaesthesia only using propofol [24,158]. In human studies, comparing TIVA with a volatile anaesthetic demonstrated that the number of CD3+, CD4+ and CD8+ T-lymphocytes decreased less following TIVA [159] and those who receive propofol-based TIVA during cancer surgery have higher overall survival [160].

Alfaxalone (3a-hydroxy-5a-pregnane-11, 20-dione) is a neurosteroid anaesthetic that interacts with the gamma aminobutyric acid A receptor, producing anaesthesia and muscle relaxation [161]. Moreover, studies in dogs and cats suggest no beneficial analgesic effect of alfaxalone compared with propofol [162,163,164]. Studies in vitro have shown the impact of alfaxalone on canine peripheral blood lymphocyte function and did not find any influence. However, the authors of this manuscript acknowledge that the effects of various anaesthetics on a living organism could vary [131]. Studies in rats demonstrate that alphaxalone could effectively inhibit the proliferation of C6 glioma cells [165] as well as the histamine release from mast cells [166]. Due to an absence of research, the effect of alfaxalone on the immune system and on tumour cells is still unknown.

Ketamine is a phenylcyclohexylamine derivative and antagonist of the excitatory neurotransmitter N-methyl-D-aspartate (NMDA) receptors [167]. It is well known for its dissociative anaesthetic qualities, but it also has antihyperalgesic [168] and anti-inflammatory effects, described in both in human [169] and laboratory animals [170,171]. The anti-proinflammatory effect of ketamine is indeed associated with its capacity to regulate cytokines and inflammatory mediators, as well as recruit inflammatory cells. [169]. Ketamine can lessen the demand for opioids [172] and their immunosuppressive effects [173], as well as enhance morphine’s effectiveness in cancer pain management [174]. Animal studies suggest that ketamine can inhibit NK cell activity and circulating numbers, resulting in increased metastasis [91]. These findings also suggest that, in vivo, this drug can suppress the immune system via stimulation of the sympathetic nervous system, explaining why β-receptor antagonists can diminish the pro-metastatic effects of ketamine [91]. On the other hand, in vitro studies demonstrated that the effect of ketamine on the cytotoxicity of peripheral lymphocytes was insignificant [131]. On an equine macrophage cell line, ketamine inhibited lipopolysaccharide-induced TNF-α and IL-6 [175]. In a rat model, ketamine promoted lung metastasis via NK cell suppression [91] and decreased the DC production of IL-12, indicating that this drug profoundly influenced T cell differentiation [176] as well as stimulated the development of metastases in lung carcinomas [151]. Human studies report that low preoperative doses of ketamine maintained IL-2 expression, which is crucial for preventing autoimmunity and T-cell differentiation, but once pain is a suppressor of IL-2 release, it is unclear as to whether this result was a direct effect or a consequence of the analgesic effect [177]. Moreover, ketamine causes the apoptosis of T lymphocytes [178] and inhibits the functional maturation of dendritic cells [176]. They also block the NMDA receptor to promote anti-tumour actions [179] as well as inhibit the differentiation of monocytes into immature DCs and the beta TGF-dependent process [180].

## 6. Conclusions

The influence of surgery and anaesthetic agents on the immune system and tumour cells is still uncertain. Despite several studies, the controversial outcomes remain a fact and can be attributed to distinct experimental conditions, such as in vitro or in vivo research, the type of cell line, surgical technique, and anaesthetic agents and their concentration. Moreover, we know that the perioperative period is a time when the body is more susceptible to developing a compromised immune response and, consequently, an increased risk of tumour dissemination. However, this time can be seen as an opportunity to control or minimise the impact of anaesthetic and surgical effects. After reviewing the above studies, propofol-based anaesthesia appears to have immunoprotective and anti-tumour effects and has been shown to decrease surgical stress, immunosuppression and angiogenesis. On the other hand, inhaled anaesthetics have been associated with a higher rate of tumour recurrence and metastasis because of their capacity to induce pro-inflammatory activity and decrease immune function. As well, minimally invasive surgery, when compared with open surgery, shows major tissue protection and minor stress responses. However, it has been linked to prolonged surgical and anaesthetic times.

Through the new anaesthesia and surgery strategy, the perioperative period is already recognised as a window of opportunity for minimising their side effects. To draw definitive conclusions about the outcomes of surgery and anaesthesia in oncology patients, additional clinical trials are required.

## Data Availability

The data supporting the findings of this study are shown in the manuscript.
